# Study of Polyphenol Content and Antioxidant Capacity of *Myrianthus Arboreus* (Cecropiaceae) Root Bark Extracts

**DOI:** 10.3390/antiox4020410

**Published:** 2015-06-09

**Authors:** Pierre Betu Kasangana, Pierre Selim Haddad, Tatjana Stevanovic

**Affiliations:** 1 Département des Sciences du Bois et de la Forêt, Faculté de Foresterie, de Géographie et de Géomantique, Université Laval, Québec, QC G1V 0A6, Canada; E-Mail: pierre-betu.kasangana.1@ulaval.ca; 2 Département de Pharmacologie, Faculté de Médecine, Université de Montréal, Montréal, QC H3T 1J4, Canada; E-Mail: pierre.haddad@umontreal.ca; 3 Institut sur la Nutrition et des Aliments Fonctionnels (INAF), Québec, QC G1V 0A6, Canada

**Keywords:** *Myrianthus arboreus*, phenolic content, antioxidant activity, DPPH, *in vitro* phosphomolybdenum, ORAC method

## Abstract

In order to evaluate the therapeutic potential of polyphenolic extracts from root bark of *M. arboreus*, we have determined the content of various polyphenols in aqueous and ethanol (EtOH) extract as well as two sub-fractions of the latter: ethyl acetate (EAc) and hexane (Hex). The total phenols, flavonoids, hydroxycinnamic acids and proanthocyanidins have been determined for all studied extracts/fractions by spectrophotometric methods. Both TP content (331.5 ± 2.5 mg GAE/g) and HCA content (201 ± 1.5 mg CAE/g) were determined to be the highest in EAc fraction of EtOH extract. All studied extracts were however determined to have a low content in flavonoids. The determination of antioxidant capacities of the studied extracts has also been performed by the following *in vitro* antioxidant tests: DPPH scavenging, phosphomolybdenum method and oxygen radical absorbance (ORAC_Fl_ and ORAC_PRG_) assay. The results of the DPPH free radical and ORAC_Fl_ assays showed that there is no significant difference between the EAc fraction and Oligopin^®^, but the EAc fraction exhibited the highest antioxidant capacity as determined by the phosphomolybdenium method. In addition, the EtOH extract was determined to have the same antioxidant efficiency as the synthetic antioxidant BHT or commercial extract Oligopin^®^ by phosphomolybdenum method. On the other hand, a positive correlation (*r* < 0.6) was found between different classes of polyphenols and the results of the phosphomolybdenum method, ORAC_Fl_ as well as ORAC_PRG__,_ except for the DPPH assay, for which a negative correlation was indicated (*r* < 0.62). Interestingly, it seems that the content in hydroxycinnamic acids played a big role in all assays with *r* < 0.9. According to the present study, EAc fraction and EtOH extract should be further studied for the potential use in the pharmaceutical and food industry.

## 1. Introduction

In recent decades, natural plant products have been the object of growing interest because of their valuable therapeutic properties. Available data indicate that natural products have beneficial effects on human health, notably in relation to their antioxidant activity. This property is particularly important for phenolic compounds because of their ability to scavenge free radicals originating from different oxygen and nitrogen species (ROS/RNS), such as superoxide anion (O_2_^•−^), hydroxyl radical (HO^•^) peroxyl radical (ROO^•^), nitric oxide (NO^•^), hypochlorite ion (ClO^•−^), hydrogen peroxide (H_2_O_2_), singlet oxygen (^1^O_2_) and peroxynitrite (ONOO^•−^) [1,2,3,4]. For most living organisms, ROS are produced continuously during normal physiological events but their overproduction can affect and damage essential biomolecules such as nucleic acids, lipids, proteins and carbohydrates. ROS can also easily initiate the peroxidation of membrane lipids, which leads to the accumulation of lipid peroxides [1]. All organisms have antioxidant defenses, including antioxidant enzymes (SOD, catalase and peroxidase) and dietary components (Vitamin E, Vitamin C and beta-carotene) that are used to remove or repair damaged molecules [1,4,5]. If ROS are not trapped or eliminated by such cellular constituents, they may lead to serious diseases. According to Halliwell *et al.* [4], ROS have been associated with over 100 diseases, including Alzheimer’s, cancer, atherosclerosis, diabetes mellitus, hypertension and aging [3,6].

Antioxidants are thus useful in protecting cells from such oxidative damage. Synthetic antioxidants, such as butylated hydroxyanisole (BHA) and butylated hydroxytoluene (BHT) are often used as food additives. However, they have been associated with liver damage and carcinogenesis [4,5,7]. Natural antioxidants are an interesting alternative in view of their variety of structures and chemical interactions, as well as the numerous biological activities they can perform. Intensive research activities are currently being carried out on plant antioxidants to meet this challenge. Indeed, several studies have reported that polyphenols, such as flavonoids, hydroxycinnamic acids and proanthocyanidins, act as powerful antioxidants [3,6,8]. These are widely distributed in the plant kingdom and particularly in forest trees, The flavonols (e.g., quercetin) and hydroxyicinnamic acids (e.g., caffeic and ferulic acids) were determined to be more potent antioxidants than ascorbic acid. Phenolic antioxidants have been recognized as an important class of food ingredients and are currently added to various food products in order to provide additional health benefits [5,8].

Abundant and low-value raw materials such as residues of wood transformation represent a challenging, yet highly promising, source in which to find new natural, safe and economical antioxidant substances, especially in relation to the concept of sustainability [2,9]. Recent studies have reported that natural antioxidant molecules found in the bark of certain trees can inhibit ROS and thus contribute to the health benefits of the forest biomass [10,11].

In this present study, we sought to determine whether the extracts from the root bark of an indigenous forest species from Africa represent a good source of antioxidants. *Myrianthus arboreus* P. Beauv. (Cecropiaceae) is a small tree growing in tropical regions of Africa. The root bark aqueous extract is traditionally used in Congo to treat diabetes [12]. Previous work on *M. arboreus* reported the isolation of peptide alkaloids from leaves and of many pentacyclic triterpenes from the root wood [13]. Among those compounds, ursolic and arjunolic acids were found to exhibit antioxidant activity as well as metal chelating properties. This was attributed in part to the two hydroxyl groups that they contain [14,15]. However, the root bark of the plant has not yet been subject to serious scientific study. We wished to evaluate the potential health benefits of polyphenolic extracts from this part of *M. arboreus*. We therefore assessed the antioxidant and radical scavenging capacities of aqueous and ethanol (EtOH) extracts as well as two sub-fractions of the latter, namely ethyl acetate (EAc) and hexane (Hex). The antioxidant capacities of these extracts have been determined by the following *in vitro* antioxidant tests: DPPH scavenging, phosphomolybdenum method and oxygen radical absorbance capacity (ORAC_Fl_ and ORAC_PRG_). Moreover, the total content of phenols, flavonoids, hydroxycinnamic acids and proanthocyanidins has been determined by spectrophotometric methods for all studied extracts/fractions.

## 2. Experimental Section

### 2.1. Material Sampling

The root bark of *Myrianthus arboreus* was collected in the city of Bas-Congo (R.D. Congo), from Jardin botanique de Kisantu, in December 2012. One root bark specimen was identified by Jean-Pierre Habbari and kept in the herbarium of the Department of Sciences in the University of Kinshasa. After air-drying, the root barks were packed to avoid exposition to water or any other contaminant and sent from Kinshasa (R.D. Congo) to Laval University (Quebec, Canada). They were then ground into powder with a hammer mill and sieved with as 40–60 mesh. The crude powder was then stored at −4 °C in darkness prior to the experiments. This crude powder was used to prepare aqueous (AQ) and ethanol (EtOH) extracts as well as two sub-fractions of the latter: ethyl acetate (EAc) and hexane (Hex) sub-fractions.

### 2.2. Extraction and Separation of Sub-Fractions

The hot aqueous extract was obtained using 15 g of dry ground material extracted with 150 mL of distilled water at 85 °C under reflux conditions for 2 h, according to procedure developed by Diouf *et al.* [16]. The extract was filtered through a Whatman No.41 filter paper and rinsed with 75 mL of hot distilled water. The aqueous filtrate (225 mL) was freeze-dried to yield crude hot water extract (AQ). All extracts were stored at −20 °C prior to analyses.

The ethanol extract was prepared with 95% ethanol, as described by St-Pierre *et al.* [17], using the same ratio of dry bark/solvent as for water, by continuous shaking (250 rpm) in an orbital shaker (Barnstead Lab-Line model 4633 , Melroso Park, IL, USA) at room temperature for 24 h. The extract was then filtered through Whatman No. 41 paper and washed with 75 mL of 95% ethanol. The filtrate (225 mL) was rotary-evaporated under vacuum at 40 °C to remove ethanol, and then freeze-dried to obtain the crude ethanol extract (EtOH).

The EtOH extract was further separated into two sub-fractions, namely hexane (Hex) and ethyl acetate (EAc) fractions. One gram of EtOH extract was dissolved in 50 mL of distilled water and transferred into 100 mL separating funnel. The extract was sequentially partitioned with (3 × 25 mL) hexane and then with ethyl acetate to obtain two different sub-fractions. Each sub-fraction (75 mL) was concentrated to dryness in vacuo at 40 °C using a rotary evaporator and stored in amber vials at −20 °C prior to analyses.

### 2.3. Chemical Composition of Crude Extracts

#### 2.3.1. Total Phenol Content

The total phenol content of the *M. arboreus* extracts and sub-fractions was measured spectrophotometrically according to the Folin-Ciocalteu’s method, as described by Scalbert *et al.* [18]. Dry extracts were solubilized with methanol to a final concentration of 200 μg/mL. Aliquots of these samples (0.5 mL) were mixed with 2.5 mL of the Folin–Ciocalteu reagent (diluted 10 times with distilled water) and 2 mL of an aqueous sodium carbonate solution (75 mg/mL). The final mixture was heated at 50 °C for 10 min, after which time the absorbance was read at 760 nm against a blank (solution with no extract added). Gallic acid (Sigma-chemical, St. Louis, MO, USA) was used to prepare a calibration curve and results are expressed in gallic acid equivalents (mg∙GAE/g dry extract). 

#### 2.3.2. Flavonoid Content

The content in flavonoids was determined spectrophotometrically according to the AlCl_3_ method developed by Brighente *et al.* [19]. Dry extracts were solubilized with methanol to a final concentration of 1 mg/mL. Aliquots of these samples (2 mL) were mixed with an equal volume of 2%∙w/v AlCl_3_·6H_2_O solution. The mixture was vigorously shaken and absorbance was read at 415 nm after 1 h of incubation at 20 °C. Quercetin (Sigma-chemical, St. Louis, MO, USA) was used to prepare a calibration curve and results are expressed as quercetin equivalent (mg∙QE/g dry extract).

#### 2.3.3. Hydroxycinnamic Acids Content

The content in hydroxycinnamic acids (HCA) was determined according to the procedure described by St-Pierre *et al.* [17]*.* Dry extracts were solubilized with methanol to a final concentration of 1 mg/mL. Aliquots of these samples (0.5 mL) was added to 1 mL of 0.5 M HCl, 1 mL of Arnow reagent (10%∙w/v sodium nitrate and 10%∙w/v sodium molybdate in distilled water), 1 mL of 2.125 M NaOH and 1.5 mL of distilled water. The mixture was thoroughly shaken and the absorbance was read at 525 nm. Each solution was compared to a blank containing all but the Arnow reagent (replaced by distilled water). Chlorogenic acid (Sigma-chemical, St. Louis, MO, USA) was used to prepare a calibration curve and results are expressed as chlorogenic acid equivalents (mg∙CAE/g dry extract).

#### 2.3.4. Proanthocyanidins Content

The content of proanthocyanidins was assessed following the method developed by Porter *et al.* [20]*.* Dry extracts were solubilized with methanol to a final concentration of 1 mg/mL. Aliquot of these samples (1 mL) was added to 6 mL of 19:1 *n*-butanol:HCl. Then, 0.2 mL of 2% ferrous ammonium sulfate (FeNH_4_(SO_4_)_2_) in 2 M HCl was added. The mixtures were shaken by a vortex mixer and heated to 95 °C in closed glass tubes in a water bath for 50 min. They were then put in icy water for 10 min and left for 5 min on the bench top to bring them to room temperature. Their absorbance was read at 550 nm against a blank. Cyanidin chloride (Indofine Chemical Co., Hillsborough, NJ, USA) was used to prepare a calibration curve and results are expressed as cyanidin chloride equivalents (mg CCE/g dry extract).

### 2.4. Evaluation of Antioxidant Activity

#### 2.4.1. Total Antioxidant Capacity (TAC)

The total antioxidant capacity (TAC) of *M. arboreus* extracts and fractions was spectrophotometrically determined by the phosphomolybdenum assay using the method described by Prieto *et al.* [21]. Briefly, 0.3 mL of a 1 mg/mL extract solution in methanol was mixed with 2.7 mL phosphomolybdenum reagent (28 mM sodium phosphate and 4 mM ammonium molybdate in 0.6 M sulphuric acid) in capped test tubes. Incubation was then carried out for 90 min in a water bath at 95 °C. After cooling to room temperature, the absorbance of the solutions was measured using a UV-visible spectrophotometer (Varian Inc., Mulgrave, Australia) at 695 nm against a blank (0.3 mL methanol without plant extract). TAC results were expressed as Trolox (Sigma-Aldrich, St. Louis, MO, USA) equivalents (mg TE/g of dry sample). Butylated hydroxytoluene (BHT; Sigma-Aldrich, St. Louis, MO, USA) and Oligopin^®^ (DRT nutraceutical, Vielle-St-Girons, France) were used as reference controls.

#### 2.4.2. Free Radical Scavenging of DPPH Radical

The free radical-scavenging activity of *M. arboreus* extracts and fractions were evaluated according to the method described by Wang *et al.* [22], with some modifications. Briefly, equal volumes of the methanolic solution of the 1,1-diphenyl-2-picrylhydrazyl (DPPH) free radical (200 μM) and of the extracts (at the various concentrations indicated) were mixed in a rapid kinetic accessory SF-22 (Hi-Tech Scientific, Salisbury, UK). The temperature was kept at 30 °C. The radical scavenging effect was continuously followed by monitoring the change of absorbance at 516 nm for 30 min, against a methanol solvent blank.

The scavenging percentage was calculated according to:

% Scavenging = (A_control_ – A_sample_)/A_control_ × 100 (1)

where A_control_ is the absorbance at 516 nm of 100 μM DPPH solution without addition of the extract/fractions, A_sample_ is the absorbance at 516 nm of 100 μM DPPH with 5–100 μg/mL of sample. Trolox was used as a reference standard. BHT and Oligopin^®^ were also used for comparison. EC_50_ value (concentration of extract necessary to reduce by 50% the initial quantity of DPPH) was determined by a graph plotting percentage inhibition against concentration.

#### 2.4.3. Oxygen Radical Absorbance Capacity (ORAC) Assay

ORAC values of *M. arboreus* extracts and fractions have been determined using two target compounds namely fluorescein (ORAC_Fl_) and pyrogallol red (ORAC_PRG_). The consumption of the probe molecules, fluorescein or pyrogallol, associated to its incubation in presence of 2,2′-azo-bis(2-amidinopropane)dihydrochloride (AAPH) was estimated from fluorescence (f) and absorbance (A) measurements, respectively.

##### 2.4.3.1. Orac-Fluorescein

The ORAC-Fl activity of extracts/fractions was measured automatically using a Galaxy fluorimeter (BGM Labtech, Durham, NC, USA). Prior to analysis, the extracts and fractions were dissolved in a solvent mixture of acetone/water/acetic acid (70:29.5:0.5 AWA), according to Prior *et al.* [23]. Then, 200 μL of a fluorescein solution (0.036 mg/L) and 20 μL of the diluted samples were added in wells. Seventy-five microliters of a 2,2-azobis-2-aminopropane dihydrochloride (AAPH) solution (8.6 mg/L) was injected automatically after four cycles from the incubator held at 37 °C. AAPH was used as a source of the peroxyl radical, which is generated as a result of the spontaneous decomposition of AAPH at 37 °C. The analyzer was programmed to record the fluorescence, at the emission wavelength of 520 nm and excitation wavelength of 485 nm, 35 times during the 120 min of analysis. ORAC_Fl_ results were calculated by using a quadratic regression equation (*y* = 0.21 *x* + 3.37; *R*^2^= 0.99) obtained from concentrations of the Trolox reference standard and net area under the fluorescein decay curve. The results are expressed as micromoles of Trolox equivalents (TE) per gram (mmol of TE/g).

The area under curve (AUC) was calculated as:

AUC = (1 + f_5_/f_4_ + f_6_/f_4_ + f_7_/f_4_ + … + f*_i_*/f_4_) × CT (2)

where f_4_ = fluorescence reading at cycle 4, f*_i_* = fluorescence reading at cycle I, and cycle time in minutes. The relative ORAC value (Trolox equivalent) was calculated as

ORAC value = [(AUC_sample_ – AUC_blanck)_/(AUC_reference_ – AUC_blanck_)] × F [reference] (3)

where: F = dilution factor and [reference] = Trolox millimolar concentration

##### 2.4.3.2. ORAC-Pyrogallol

Peroxyl radical scavenging activity was determined by the method described by López-Alarcón *et al.* [24] with slight modifications. Stock solutions of pyrogallol (300 μM) were prepared daily in phosphate buffer/ethanol, 70/30 at pH 7.0. Briefly, 20 μL of the *M. arboreus* extract/fraction solution (0–1.0 mg/mL; in a mixture of water/methanol 1/1) were mixed with 200 μL of a 30 μM solution of pyrogallol red solution at 37 °C in the heat-controlled cuvette of a UV-visible spectrophotometer. Then, 5 μL of a 600 mM AAPH (2,2′-azo-bis (2-amidinopropane hydrochloride) solution was added to initiate the reaction. Pyrogallol red consumption was measured spectrophotometrically by monitoring the decrease of solution absorbance (A) at 540 nm. Values of A/A0 were plotted as a function of time. Integration of the area under the curve (AUC) was performed up to a time such that A/A0 reached a value of 0.2. The AUC was calculated as:

AUC = (1 + A1/A0 + A2/A0 + A1/A0 + … + A*i*/A0) (4)

where A0 is the initial absorbance reading at 0 min and Ai is the absorbance reading at time *i*. The relative ORAC value (Trolox equivalent) was calculated according to the following equation: *y* = 0.12 *x* − 0.97; *R*^2^ = 0.99.

### 2.5. Statistical Analysis

The results are expressed as the mean of three measurements ± standard deviation. The ANOVA variance analysis was applied to compare the means followed by the Tukey test. The statistical significance threshold was set at *p* < 0.05. Descriptive statistical analysis was performed using Microsoft Excel and/or GraphPad Prism version 5.01 (GraphPad Software Inc., La Jolla, CA, USA). The non-parametric Spearman’s correlation test was used for correlations (*p* < 0.05).

## 3. Results and Discussion

### 3.1. Content of Polyphenols

Phenolic compounds are ubiquitous constituents of plants and their major sources in human diet are fruit, vegetables and various beverages. The same bioactive polyphenols, such as flavonoids, proanthocyanidins and derivatives of hydroxycinnamic acids, are also available from forest trees and consequently from the residues of industrial wood transformation [5,8,10]. In order to evaluate the potential antioxidant capacity of the extracts from root bark of *M. arboreus*, it was reasonable to determine the content of various polyphenols in aqueous extract (AQ), in ethanol (EtOH) extract and in two sub-fractions of the latter:ethyl acetate (EAc) and hexane (Hex). The total phenols, flavonoids, proanthocyanidins and hydroxycinnamic acids have been determined for all studied extracts/fractions by spectrophotometric methods and the results are presented in Table 1.

**Table 1:** Total phenols (TP), Total flavonoids (TFv), Proanthocyanidins (PAs) and Total hydroxycinnamic acids (THCA) of the extracts and ethanolic fractions.

Extracts	Yields (%)	TP (mg∙GAE/g)	TFv (mg∙QE/g)	PAs (mg∙CChE/g)	THCA (mgCAE/g)
AQ	10 ± 0.38	146.6 ± 6.5 ^b^	2.9 ± 0.1	6 ± 0.4 ^a^	31 ± 1.5 ^a^
EtOH	2.3 ± 0.1	292.2 ± 1.3 ^c^	3.6 ± 1.3	71.3 ± 3.3 ^c^	173 ± 1.3 ^c^
EAc	33.9 ± 1	331.5 ± 2.5 ^d^	5.3 ± 0.1	68.3 ± 0.8 ^c^	201 ± 1.5 ^d^
Hex	60 ± 3	138 ± 1.6 ^a^	2.4 ± 0.1	26.5 ± 0.7 ^b^	58 ± 2.6 ^b^
Oligopin^®^		572.9 ± 12.1 ^e^	7.4 ± 0.1	105 ± 9.6 ^d^	335.5 ± 3.4 ^e^

Different letters indicate significantly different results according to Tukey’s test at 95% confidence level. Yields were calculated in reference to initial raw material for AQ and EtOH extracts, whereas yields of EAc and Hex are expressed in reference to the parent EtOH extract. TP = total phenols content expressed in mg∙of gallic acid equivalents (GAE) per g of dry extracts; TFv = total flavonoid content expressed in mg of quercetin equivalents (QE) per g of dry extracts; PAs = proanthocyanidin content in mg of cyanidin chloride equivalents (CChE) per g of dry extracts; THCA = total hydroxycinnamic acids content expressed in mg of chlorogenic acid equivalents (CAE) per g of dry extracts.

Among the studied extract/fractions, the highest total phenols content was found in the EAc fraction (331.5 ± 2.5mg∙GAE/g) that also contained the highest hydroxycinnamic acids content (201 ± 1.5 mg∙CAE/g). In contrast, all studied extracts were low in flavonoids content. Proanthocyanidins (~70 mg∙CChE/g) content was similar in the EtOH extract and its EAc fraction. The comparison of *M. arboreus* extracts/fractions with Oligopin^®^ demonstrates that the latter contains higher amounts of all studied classes of polyphenols. Interestingly, however, the EAc fraction contained levels of flavonoids and hydroxycinnamic acids that approached best those determined for Oligopin^®^. Oligopin^®^ is used as reference because it is a polyphenol-rich extract prepared by a standardized extraction of Maritime pine bark with water. Interestingly, this chemical analysis of *M. arboeus* revealed a content of hydroxycinnamic acids, both in EtOH extract and its EAc fraction, that is superior to that of ethanol extracts of bark and wood of Canadian species, such as such as sugar maple (91 ± 60 mg∙CAE) [17] or yellow birch (117 ± 10 mg∙CAE) [25]. The high content in hydroxycinnamic acids thus provides value to the root bark extracts of *M. arboreus* and encourages further studies to develop this value.

### 3.2. Antioxidant Capacities Determined by Different Methods

#### 3.2.1. DPPH Radical Scavenging Activity

The DPPH assay measures the hydrogen-donating antioxidant (AH) activity of tested substances. The scavenging effect of *M. arboreus* extracts and standards (BHT and Oligopin^®^) were thus assessed and expressed in terms of the concentration of extract necessary to reduce by 50% the initial quantity of DPPH. Lower EC_50_ values thus indicate a higher DPPH free radical scavenging activity.

**Figure 1:**
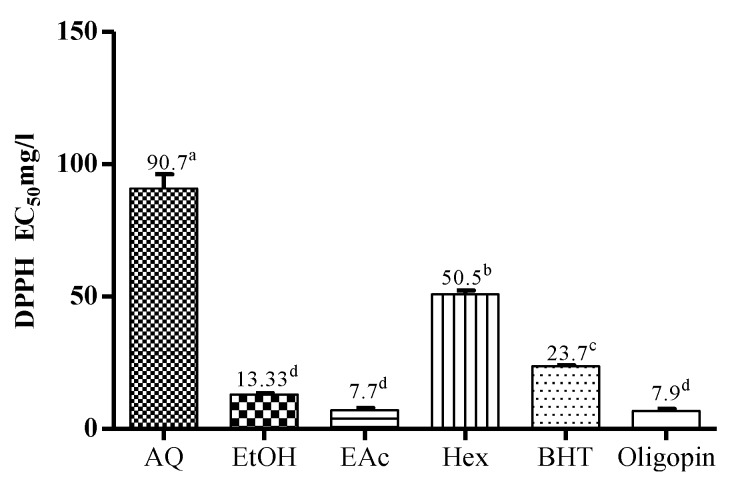
Radical scavenging activities of *M. arboreus* extracts/fractions determined by the reduction of DPPH free radical. The extracts having the same letter present no significant differences (*p* < 0.05) according to Tukey statistical test.

As shown in Figure 1, the EC_50_ value of the EtOH extract (13.3 μg/mL) and its EAc fraction (7.7 μg/mL) demonstrate a radical scavenging activity better than that for the reference chemical antioxidant BHT (23.7 μg/mL). This result is very similar to those obtained for several woody-plant extracts in comparison to BHT [16,25] but also compared to Vitamins E and C, other antioxidants commonly used by the food industry [3,5]. Reported EC_50_ values for BHT depend on the method used in various laboratories. For example, an EC_50_ value of 150 μg/mL for BHT was obtained by Kong *et al.* [26], whereas the value that we obtained is comparable to that published by Misha *et al.* [27]. On the other hand, the DPPH radical scavenging activities of EtOH extract and EAc fraction compared favorably with Oligopin^®^ (7.9 μg/mL). The descending rank order scavenging capacity of *M. arboreus* extracts/fractions was as follows: EAc (7.7μg/mL) ≈ EtOH (13.33 μg/mL) > Hex (50.5 μg/mL) > AQ (96.11 μg/mL).

#### 3.2.2. Total Antioxidant Capacity (TAC) by Phosphomolybdenum Method

**Figure 2:**
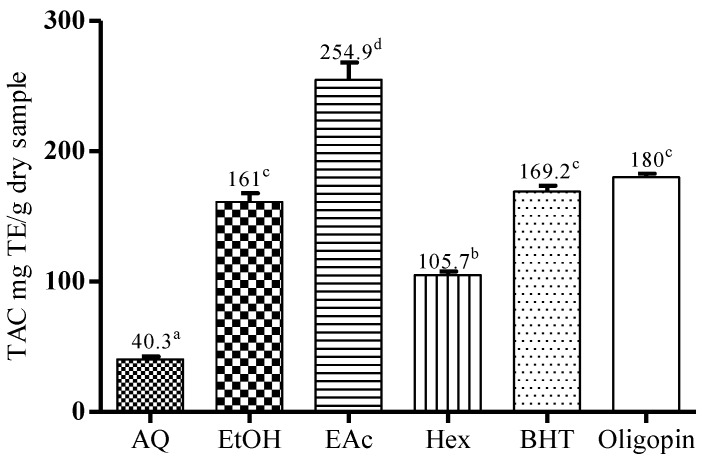
Total antioxidant capacity of *M. arboreus* AQ extract, EtOH extract and its fractions. The extracts having the same letter present no significant differences (*p* < 0.05) according to Tukey statistical test.

Figure 2 presents the total antioxidant capacity obtained through the phosphomolybdenum assay for each extract in comparison with that of BHT (synthetic antioxidant) and of Oligopin^®^, both used as references. Overall, the aqueous (AQ) extract (40.3 ± 3.9 mg TE/g) showed a much lower antioxidant potential than other extracts and fractions as well as standards BHT (169.2 ± 7.8 mg TE/g) or Oligopin^®^ (180.8 ± 1.9 mg TE/g). This result is consistent with the lowest antiradical effect of AQ extract determined by the DPPH assay. In contrast, the EAc fraction (254.9 ± 23.1 mg TE/g) was the best antioxidant as demonstrated by the highest value of TAC compared to other extracts and standards used. It is followed by the EtOH extract (161.1 ± 11.9 mg TE/g), which is not significantly different from BHT and Oligopin^®^. The antioxidant capacity of the EAc fraction may well be related to the proportion of phenolic compounds that constitute it. 

#### 3.2.3. Oxygen Radical Absorbance Capacity (ORAC) Assay

The ORAC has been widely employed to evaluate the antioxidant capacity of beverages [23], food and plant extracts. This assay measures the protection conferred by an antioxidant to a target molecule, which is oxidized by the peroxyl radical, 2,2′-azo-bis(2-amidinopropane) dihydrochloride (AAPH). Originally, ORAC was proposed by Cao *et al.* [28] and used beta-phycoerythrin as the target molecule. Since 2005, beta-phycoerythrin is not encountered in publications; it has been replaced by fluorescein as a probe, since the latter is photostable and does not interact with polyphenols [29]. ORAC_Fl_ values are mainly influenced by the content of phenolic compounds in a plant extract [6,24]. That is why this method is often completed by the ORAC_PRG_ test. Pyrogallol Red is a color reagent easily oxidized by bromate, iodate and hydrogen peroxide; it is used for the study of antioxidant trapping properties. ORAC_PRG_ provides more information about the ability of a given sample to reduce the oxidative damage caused to valuable molecules by peroxyl radicals. Each of these methods helps to evaluate the antioxidant properties of a complex mixture. Lopez-Alarcon *et al.* [24] estimated that the ratio ORAC_PRG_/ORAC_Fl_ can be considered as an indication of the average quality of antioxidants. In studies using ORAC_Fl_, it is possible to evaluate lipophilic and hydrophilic antioxidant components of extracts. However, several studies have reported that hydrophilic ORAC_Fl_ is responsible for >90% of the total antioxidant capacity of the sample [30]. For that reason, we are reporting here only the hydrophilic ORAC_Fl_ results obtained for the *M. arboreus* extracts/fractions.

##### 3.2.3.1. ORAC-Fluorescein Values

**Figure 3:**
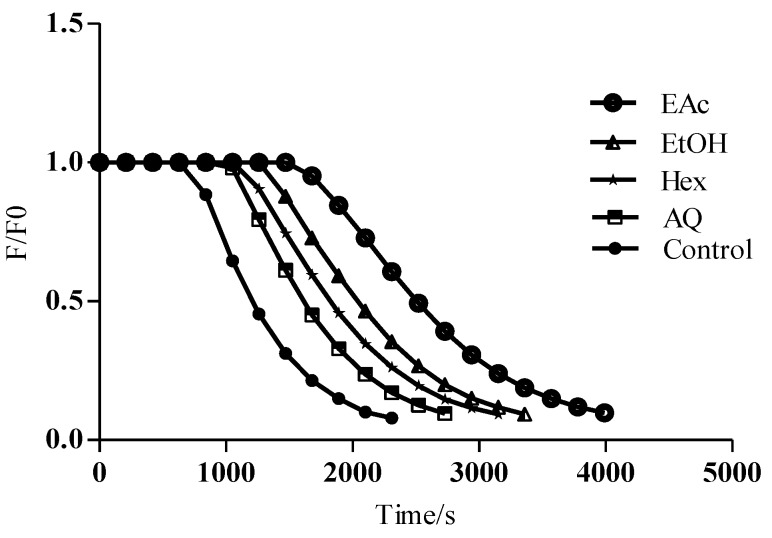
Kinetic profiles of fluorescein consumption in the presence and absence of *M. arboreus* extracts and its fractions. Fluorescein (70 nM) was incubated in the presence of AAPH (10 mM) and different amounts of a *M. arboreus* as described in Materials and Methods.

The results of AAPH-mediated oxidation of fluorescein in the absence and in the presence of extracts/fractions of *M. arboreus* are presented in Figure 3. In the absence of the extract, a gradual decrease in fluorescein fluorescence was observed with an almost total consumption after 30 min of incubation. The addition of different *M. arboreus* extracts produced a lower consumption rate and the induction time depended on the extract/fraction used. The ORAC_Fl_ values obtained are presented in Table 2. Among the four extracts of *M. arboreus* studied, the highest ORAC_Fl_ value was found for EAc fraction (9.14 ± 0.9 mmol of TE/g) and the lowest for the AQ extract (1.47 ± 0.13 mmol of TE/g) and Hex fraction (1.60 ± 0.15 mmol of TE/g). As determined in previous tests, the EtOH extract (4.82 ± 0.24 mmol of TE/g) followed the EAc fraction. This shows that the EAc fraction and EtOH extract are very effective and demonstrate high antioxidant potential by this assay. In fact, the EAc sample was similar to Oligopin^®^ (12.2 ± 5 mmol of TE/g). The ORAC value of Oligopin^®^ that we obtained is close to 15 mmol of TE/g, a value estimated by the food industry as well as similar studies to prove the antioxidant potential of this commercial extract [30,31]. In this sense, the *M. arboreus* EAc fraction, which is rich in polyphenols, should again arouse interest for the evaluation of its potential health benefits.

**Table 2:** ORAC_PRG_, ORAC_Fl_ and ORAC_PRG_/ORAC_Fl_ ratio values of *M. arboreus* extract and fraction.

Extracts	ORAC_Fl_ (mmol of TE/g)	ORAC_PGR_ (mmol of TE/g)	Ratio ORAC_PGR_/ORAC_Fl_
AQ	1.47 ± 0.1 ^a^	0.35 ± 0.02 ^a^	0.24
EtOH	4.82 ± 0.2 ^a^	1.3 ± 0.05 ^c^	0.27
EAc	9.14 ± 0.9 ^b^	2.2 ± 0.09 ^d^	0.24
Hex	1.60 ± 0.2 ^a^	0.5 ± 0.06 ^b^	0.32
Oligopin^®^	12.2 ± 2.7 ^b^	4.3 ± 0.07 ^e^	0.35

Means with different letters in the same column are significantly different at *p* < 0.05(ANOVA, followed by Tukey’test.

##### 3.2.3.2. ORAC-Pyrogallol Red values

**Figure 4:**
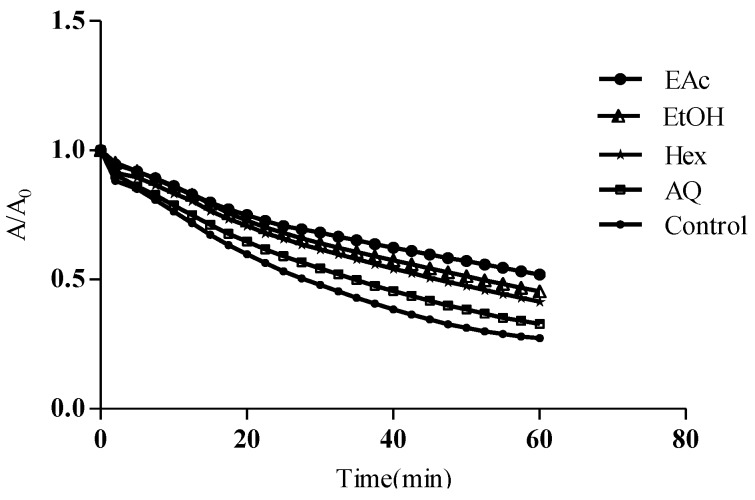
Kinetic profiles of pyrogallol consumption in the presence and absence of *M. arboreus* extracts/its fractions. Pyrogallol (30 μM) was incubated in presence of AAPH (600 mM) and different amounts of *M. arboreus* products as described in Materials and Methods.

In ORAC_PRG_, pyrogallol red and AAPH were used as the sources of target molecule and peroxyl radical, respectively. As shown in Figure 4, a reduced consumption rate of pyrogallol red was observed when *M. arboreus* extracts/fractions were added to the solution. Under control conditions, a gradual decrease in pyrogallol absorbance was observed with consumption reaching about 70% after 60 min of incubation, which is consistent with the kinetic profile of consumption of PGR obtained by Lin *et al.* [32]. Results presented in Table 2 indicate the EAc fraction was the best antioxidant with the highest ORAC_PRG_ value (2.2 ± 0.09 mmol TE/g), followed by the EtOH extract (1.3 ± 0.05 mmol TE/g). All of the extracts of *M. arboreus* studied were significantly less effective to scavenge peroxyl radical (ROO) than Oligopin^®^ (4.34 ± 0.07 mmol TE/g). In this assay, we find that the value ORAC_PRG_ of EAc fraction is almost half that of the Oligopin^®^. This result differs from those presented for previous assays, namely DPPH, TAC and ORAC_Fl_.

### 3.3. Correlations between the Content in Various Polyphenols and Antioxidant Capacities

The antioxidant activity of an extract or compound is often associated with their redox proprieties, which allow them to act as reducing agents [16,33]. Phenolic compounds constitute one of the major groups of phytochemicals acting as free radical scavengers and antioxidants [5]. The antioxidant activities of the *M. arboreus* AQ extract and EtOH extract as well as its two fractions (Hex and EAc) were measured by four methods: DPPH assay, phosphomolybdenum assay and oxygen radical absorbance capacity (ORAC_Fl_ and ORAC_PRG_) assay. EAc fraction and EtOH extract were found to have an appreciable level of antioxidant activity in different assays, when compared to the commercial extract of maritime pine bark Oligopin^®^ and to the synthetic antioxidant BHT (Figure 1 and Figure 2 and Table 1). Although the reactivity of EtOH extract varied between the different methods used, this extract was determined to be more efficient than the AQ extract or its Hex fraction.

**Figure 5:**
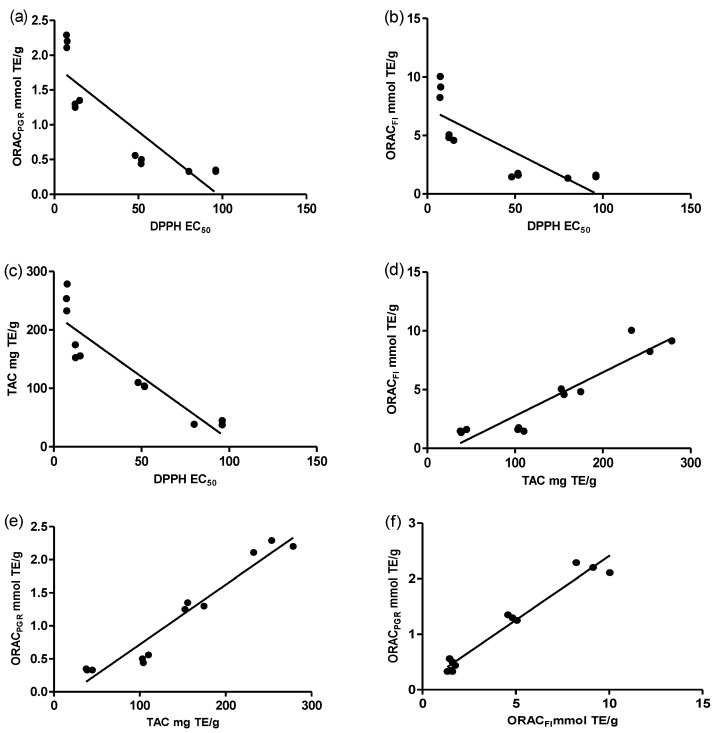
Spearman’s correlation between different antioxidant methods. (**a**) ORAC_PRG_ *vs.* DPPH (*r* = −0.94; *p* < 0.0001); (**b**) ORAC_Fl_ *vs.* DPPH (*r* = −0.86; *p* = 0.0003); (**c**) TAC *vs.* (DPPH) (*r* = −0.96; *p* < 0.0001); (**d**) ORAC_Fl_ *vs.* TAC (*r* = 0.87; *p* = 0.0002); (**e**) ORAC_PRG_ *vs.* TAC (*r* = 0.95; *p* < 0.0001); (**f**) ORAC_PRG_ *vs.* ORAC_Fl_ (*r* = 0.84; *p* = 0.0006).

The results of the antioxidant assays were consistent among themselves and correlated with polyphenolic contents of *M. arboreus* extracts and fractions as assessed using linear regression analysis. Spearman’s correlation coefficients obtained are presented in Figure 5 and Table 3. In the TAC assay, the activities of the studied extracts/fractions are associated with their reducing properties. This allows them to react with certain precursors of peroxides, thus preventing peroxide formation [34]. Overall, the polyphenols contained in the extract/fraction act by donating electrons and reacting with free radicals to convert them into more stable products and terminate free radical chain reactions [16,34]. Interestingly, the results presented in Table 3 show a significant correlation between all phenolic classes and the TAC (*p* < 0.006). In particular, we note a strong correlation between this assay and the content in hydroxycinnamic acids (*r* = 0.93, *p* < 0.0001). This result confirms the antioxidant potential of some hydroxycinnamic acids mentioned in the literature, such as caffeic and ferulic acids [5,35]*.*

**Table 3:** Spearman’s correlation coefficients obtained between antioxidant activities and the polyphenolic composition of *M. arboreus* extracts.

	DPPH	TAC	ORAC_Fl_	ORAC_PRG_
TP	*r* = −0.74, *p* = 0,01	*r* = 0.74, *p* = 0.006	*r* = 0.89, *p* < 0.0001	*r* = 0.71, *p* = 0.002
Fv	*r* = −0.62, *p* = 0.02	*r* = 0.59, *p* =0.041	*r* = 0.72, *p* = 0.007	*r* = 0.51, NS
HCA	*r* = −0.93, *p* < 0.0001	*r* = 0.93, *p* < 0.0001	*r* = 0.97, *p* < 0.0001	*r* = 0.90, *p* < 0.0001
PAs	*r* = −0*.*72, *p* = 0.002	*r* = 0.78, *p* = 0.003	*r* = 0.84, *p* < 0.0005	*r* = 0.73, *p* = 0.006

Furthermore, the results obtained in Figure 2 revealed that the EtOH extract and its EAc fraction can effectively scavenge the DPPH radical. This activity may be attributed to their ability to provide hydrogen to DPPH free radicals in order to stabilize them [5,16,25,34]. The importance of this assay lies in the fact that the free radical DPPH is comparable to the superoxide (O_2_^•−^) and hydroxyl (HO^•^) species, which are mainly responsible for the oxidative damage to biological systems [16,25,36]. Overall, Table 3 shows relatively good correlation (*r* > 0.62) between the DPPH scavenging capacity of extracts/fractions and their content in different polyphenolic classes. Similar to the TAC assay, the strongest correlation in the DPPH assay was observed with the content in hydroxycinnamic acids. This means that the free scavenging activity of the EtOH extract and its EAc fraction may be attributed, at least in part, to hydroxycinnamic acids contained in these extracts.

Apart from the DPPH^•^ radical, the peroxyl radical (ROO^•^) is another type of free radical species extensively studied in antioxidant assessment assays. Peroxyl radicals (ROO^•^) are commonly found in food and biological samples and they are formed during lipid oxidation chain reactions [37,38,39]. Once formed in the organism, they can attack unsaturated fatty acids transforming them into another peroxyl radical and so on, thereby damaging cellular membranes [2,20]. They have harmful effects on health and they are also often used to quantify the deterioration of foods [3,38]. ORAC_Fl_ and ORAC_PGR_ are among the most common methods used to quantify the peroxyl radical scavenging capacity of a sample *in vitro*. As shown in Table 3, there is a significant correlation (*r* > 0.7) found between the content of most polyphenolic classes (total phenols, hydroxycinnamic acids and proanthocyanidins) and ORAC_Fl_ or ORAC_PRG_, with the exception of flavonoid content. On the other hand, Table 2 shows that ORAC_Fl_ values are generally much higher than ORAC_PRG_. This difference depends on the methodology applied to inhibit the target molecule (fluorescein or pyrogallol) induced by peroxyl radicals [33]. In addition, it must be noted that ORAC_PRG_ values are influenced by the average “quality” of the antioxidants while ORAC_Fl_ ones depend essentially on the quantity of polyphenols present in the sample [6,24]. In any case, the EtOH extract and its EAc fraction can be regarded as good antioxidants.

As mentioned, antioxidant assays were generally consistent with one another as shown in Figure 5. Indeed, positive correlations were observed between the TAC and ORAC methods, whereas they were negatively correlated to the DPPH assay (*r* = −0.95, *p* < 0.0001), consistent with the report of Rosa *et al.* [35]. This indicates that an extract that has a lower DPPH EC_50_ value may have good reducing properties. We also found a good correlation between ORAC_Fl_ and ORAC_PGR_ (*r* = 0.84, *p* = 0.001; Figure 5) as previously seen by Zhang *et al.* [39] (*r* = 0.7, *p* < 0.01). In contrast, Lopez-Alarcon *et al.* [6] find that the two ORAC methods often have a poor correlation with each other. In addition, Lopez-Alarcon *et al.* [24] consider that the ratio ORAC_PRG_/ORAC_Fl_ represents a useful tool to estimate the quality of a particular antioxidant sample. Taking this into account, it becomes evident from Table 2 that the Hex fraction may also be taking part in antioxidant reactions. This assertion is consistent with the fact that non-phenolic compounds may affect the overall antioxidant capacity of an extract [5,10]. This may be the case with the Hex fraction, which could notably be enriched with bioactive triterpenes [13,14].

## 4. Conclusions

Based on the results obtained in this study, the EtOH extract of *M. arboreus* and especially its EAc fraction are the most effective antioxidants as determined through different *in vitro* assays. Indeed, these extracts/fractions were as efficient as, and sometimes better than, two standard antioxidants, namely the synthetic BHT and Oligopin^®^, a standardized proanthocyanidin-rich extract from French maritime pine bark. Moreover, the EtOH extract of *M. arboreus* and its EAc fraction compared favorably with Oligopin^®^ in terms of their total content of phenols, hydroxycinnamic acids and proanthocyanidins. It is plausible that the high antioxidant activities of *M. arboreus* extracts and fractions may be related to the ease with which the phenolic compounds they contain can release hydrogen atoms. In order to determine the future potential of these extracts in the pharmaceutical and/or food industry, further studies must be carried out to determine in greater detail their qualitative chemical composition as well as their toxicity.
